# Record linkage to enhance consented cohort and routinely collected health data from a UK birth cohort

**DOI:** 10.23889/ijpds.v4i1.579

**Published:** 2019-04-02

**Authors:** Karen Susan Tingay, Amrita Bandyopadhyay, Lucy Griffiths, Ashley Akbari, Sinead Brophy, Helen Bedford, Mario Cortina-Borja, Efrosini Setakis, Suzann Walton, Emla Fitzsimons, Carol Dezateux, Ronan A Lyons

**Affiliations:** 1 Office for National Statistics, Government Buildings, Cardiff Rd, Duffryn, Newport NP10 8XG; 2 Swansea University, Population Data Science, Medical School, Singleton Campus, Swansea SA2 8PP; 3 Health Data Research UK, Swansea University, Swansea University, Population Data Science, Medical School, Singleton Campus, Swansea SA2 8PP; 4 UCL, Great Ormond Street Institute of Child Health, 30 Guilford Street, London WC1N 1EH, UK; 5 NHS Digital, 1 Trevelyan Square, Boar Lane, Leeds, LS1 6AE; 6 Hertfordshire County Council, County Hall Pegs Lane Hertford SG13 8DQ, UK; 7 Centre for Longitudinal Studies (CLS) UCL Institute of Education University College London 20 Bedford Way London WC1H 0AL, UK; 8 Centre for Primary Care and Public Health, Barts; 9 London School of Medicine and Dentistry, Queen Mary University London

## Abstract

**Background:**

In longitudinal health research, combining the richness of cohort data to the extensiveness of routine data opens up new possibilities, providing information not available from one data source alone. In this study, we set out to extend information from a longitudinal birth cohort study by linking to the cohort child’s routine primary and secondary health care data. The resulting linked datasets will be used to examine health outcomes and patterns of health service utilisation for a set of common childhood health problems. We describe the experiences and challenges of acquiring and linking electronic health records for participants in a national longitudinal study, the UK Millennium Cohort Study (MCS).

**Method:**

Written parental consent to link routine health data to survey responses of the MCS cohort member, mother and her partner was obtained for 90.7% of respondents when interviews took place at age seven years in the MCS. Probabilistic and deterministic linkage was used to link MCS cohort members to multiple routinely-collected health data sources in Wales and Scotland.

**Results:**

Overall linkage rates for the consented population using country-specific health service data sources were 97.6% for Scotland and 99.9% for Wales. Linkage rates between different health data sources ranged from 65.3% to 99.6%. Issues relating to acquisition and linkage of data sources are discussed.

**Conclusions:**

Linking longitudinal cohort participants with routine data sources is becoming increasingly popular in population data research. Our results suggest that this is a valid method to enhance information held in both sources of data.

## Introduction

### Background

Linking detailed routine administrative data to equally detailed survey responses can add value to population health research. Routine data can provide information on downstream services, while surveys can give insights into attitudes behind why it happened. However, the path to linked cohort and routine data is sometimes complex and complicated.

Consent for linkage must be obtained, linkage must be performed securely and accurately, and the resulting linked dataset must be made available to researchers in an anonymised format. A report commissioned by the Wellcome Trust reported all three of these issues as barriers to data linkage [1]. Routine data, received from various sources such as Electronic Health Records (EHRs), education records, or local government authorities, can create large datasets of detailed coded data. Survey-based cohort studies provide important participant-reported information, often collected at multiple points over a long period, which may not be available through routinely collected data. Linking routine data with cohort studies improves the overall detail and knowledge we have about a participant, and provides the ability to validate each data source [2], which strengthens research and reduces the knowledge gap. Such linkage combines the volume of activity data from routine data sources with rich data in cohort studies on personal circumstances, behaviours and attitudes not captured in administrative or clinical data, enhancing the value of both sources for public health research.

While many cohort studies have been collecting consent for future linkage to multiple data sources [3-5], and some linkage has already been accomplished, the consenting and un-consenting populations may be demographically very different [6]. There are further concerns about whether broad consents obtained by cohort studies allow participants to be sufficiently informed about how their data will be used in the future [1].

Even assuming successful linkage and appropriate consent, several studies have reported significant delays in acquiring linked data from data owners [7, 2]. To a project with time-limited funding, such delays in acquiring the research data can be potentially catastrophic [8].

Linking cohort and routine data sources appears to be a useful endeavour given the ability of both to enhance each other, thus providing a richer research data source, but that this technique is not without methodological, ethical and technical challenges.

This article describes the experiences of linking a UK-wide longitudinal cohort study, the Millennium Cohort Study (MCS), to routine health records for consenting participants. The resulting linked datasets have been used to examine the health outcomes of childhood obesity, asthma, infections and injuries, and patterns of health service utilisation, including timeliness of immunisation in childhood [9, 10].

### Objectives

The purpose of this paper is to describe the methodological issues, successes and challenges encountered in linking routinely collected health datasets in England, Scotland and Wales to singleton births from the MCS cohort.

In particular, the paper will discuss the following research questions:

1. What routine health data sources are available to link to the consenting MCS cohort, given the project research areas?
2. What are the linkage rates for each of these data sources?
3. Are there any demographic differences between the consented, linked cohort and the full UK MCS cohort?
4. What are the challenges in acquiring and linking cohort data to routine data?

## Methods

### Study Participants

The study consisted of Millennium Cohort Study participants who were interviewed in Wales or Scotland at age seven, and whose legal parent/carer had given consent to link the cohort member’s health records at the age seven interview [3]. Linkage included only singleton members of the cohort.

### Data sources

#### Millennium Cohort Study

The Millennium Cohort Study (MCS) is a prospective, longitudinal survey of children born between 2000 and 2001 in the United Kingdom (UK). The MCS is conducted by the Centre for Longitudinal Studies (CLS) at University College London (UCL) [11]. The sample was originally drawn from Child Benefit records, as these were claimed by almost all families in the UK at the time. The study used a stratified cluster sampling design, and oversampled births to families living in disadvantaged areas, from the smaller UK countries and, in England, areas with high prevalence of ethnic minorities. The initial interview, taking place at nine months of age, recruited 18,552 families comprising 18,818 children (18,296 singletons; mean age 295.5 days [12]). During the interviews, information was collected on physical and mental health of the child and of their carers and on their family’s demographic and socio-economic background. Families were re-interviewed when the child was aged three, five, seven, 11, and 14 years and a further interview at age 17 began in 2018.

##### Consent to link other data sources

One of the objectives of the MCS was to extend the survey content using other linked data sources [2]. To this end, consent to link health and other administrative records to the survey responses was requested from carers at different sweeps. At the age 7 contact (MCS4), permission was sought to link to the child’s health records up to the child’s 14th birthday and 90.7% of parents consented.

Wording of the consent forms used at MCS4 in relation to health record linkage was developed in consultation with the then NHS Information Authority, now NHS Digital, and approved by the Northern and North Yorkshire Research Ethics Committee (Ref: 07/MRE03/32) [13].

##### Previous Data Linkage

At the first contact, when cohort members were approximately nine months old, parental or legal guardian consent was obtained to link MCS data and the child’s National Health Service (NHS) birth record and maternity episode hospital records in all four UK countries [14, 13]. Of the 18,552 parents interviewed at MCS1, 92% of mothers provided valid consent to link their health records [3, 14, 15]. Health record linkage was restricted to data relating to the pregnancy and birth of the cohort member [13]. Birth records were obtained from the National Health Service Central Register (NHSCR). Linkage was conducted using identifying information specified by the health data controller and included combinations of the child’s and mother’s names and dates of birth, father’s name, child’s sex, birthweight, and birth order (if part of a multiple birth), and name of the hospital of birth [14]. Linkage rates for England, Wales, Scotland and Northern Ireland health authorities ranged from 83% to 92%, with the highest rates in Scotland and Wales [14].

#### Routinely collected health datasets

The selection of routine datasets for linkage to the MCS was based on their availability and relevance to the research questions. In line with the consent to link, linkage was conducted based on whether the cohort member appeared in the target dataset at any point before their 14th birthday (maximum September 2015). However, as these are health datasets, absence of linkage does not indicate a failure in the linkage method. Not all children attend Emergency Department or hospital inpatient settings and, therefore, not all children linked via a health spine will appear in these datasets. The full list of datasets with years from which data are available is given in Table 1.

**Table 1: Datasets, with respective years, requested from Scotland and Wales for linkage with Millennium Cohort Study data for consented cohort members.** Observation: The maximum date for which data was requested was August 31^st^ 2015, although, for consent reasons, linkage is only conducted up to the child’s 14^th^ birthday. CHSP = Child Health Systems Programme; imms = immunization dataset.

Dataset setting	Wales	Scotland

Child Health (including immunisations)	2000-2015	2002-2015 (imms)
		2011-2015 (CHSP)
Emergency Department	2009-2015	2007-2015
Hospital inpatient	2000-2015	2000-2015
Primary Care General Practice	2000-2015	Not available

##### Child health datasets

Scotland and Wales both have unified child health datasets comprising birth, physical, developmental, and immunisation data, among other relevant information pertaining to the child’s health and wellbeing. In Scotland, this is the Child Health Systems Programme (Pre School and School) (CHSP), and the Scottish Immunisation and Recall System (SIRS) datasets, and in Wales, the National Community Child Health Dataset (NCCHD). The Scottish data collection began in 1993 for some health boards, but only became available for all Scottish health boards from 2011/12 [16]. Immunisation records have been consistently recorded in Scotland since 2002.

Given that some Scottish Health Boards did not start returning child health data until 2011, the MCS cohort may not have sufficient data for some research questions concerning child health up to age 14 years. Child health data has been collected in Wales from 2000 onwards and hence covers the entire period of MCS.

##### Emergency department datasets

Emergency Department data are collected in both Scotland and Wales. In Scotland, this is the Accident and Emergency version 2 dataset (A&E2), and in Wales the Emergency Department Data Set (EDDS). Scotland holds data from 2007, with diagnosis, injury fields, and an alcohol involvement flag added in 2010. Data collection began in major Welsh hospitals in 2009, and was extended to other emergency clinics in 2012.

Since the MCS cohort children were born in 2000 and 2001, neither emergency department datasets cover events occurring before age nine years.

##### Hospital inpatient datasets

Scotland and Wales hold hospital inpatient and day patient datasets. In Scotland, this information is available through the General Acute Inpatient and Day Case – Scottish Morbidity Record (SMR01) dataset, and in Wales through the Patient Episode Database for Wales (PEDW). Both Scottish and Welsh hospital inpatient datasets covered the period 2000-2015.

##### Primary Care General Practice datasets

In Wales, approximately 70-80% of General Practitioners (GPs) contribute linked data for sharing with researchers. This information is available through the Welsh Longitudinal General Practice (WLGP) dataset. When a practice signs up to share data, all the historical data are uploaded. For this study, GP records were available from 2000 to September 2015. The data include diagnoses, test results, and prescriptions issued, although the dispensing of these prescriptions is handled by separate pharmacy systems. Scotland does not yet have a national GP dataset.

### Data storage environment

Data were stored and accessed in the Secure Anonymised Information Linkage (SAIL) databank held at Swansea University in Wales.

### Linkage

The UK NHS is devolved across the four countries, England, Wales, Scotland and Northern Ireland, with different arrangements for supporting the provision of routinely collected health data to consented studies. Linkage is provided in Scotland by the NHS Information Standards Division (ISD), in Wales through NWIS and the SAIL Databank, and in England by NHS Digital. For the purposes of this project, data controllers in Scotland, Wales and England were approached. Northern Ireland was not included due to relatively small population size compared with the other UK countries [12].

Consent to link to health records in England was originally sought in 2009 but, due to multiple reorganisations in the NHS informatics organisation, changes in staff and differing interpretations of the wording of consent forms by individuals over time, we were unable to obtain agreement for linkage within the time frame of this study.

#### Anonymisation

Linkage for Scotland and Wales was approved by the appropriate data controllers and conducted by Trusted Third Party (TTP) NHS Information Services: The Scottish Information Services Division (ISD) and the NHS Wales Informatics Service (NWIS).

#### Linkage variables and methods

Both Wales and Scotland use a mixture of deterministic and probabilistic methods [17-19], based around a central population spine with lexical and soundex matching to account for spelling differences. For both sites, linkage specificity is >99% and sensitivity is between 95-100%, depending on the data source [17-19].

Since 1995, The National Health Services (NHS) in Wales assign a unique number, the NHS number, to all babies. In Scotland, the same function is served by the Community Health Index (CHI) number. These unique numbers populate many of the administrative health databases in the UK.

**Figure 1: Information required by health service Trusted Third Parties to create linkage identifiers for this project:**
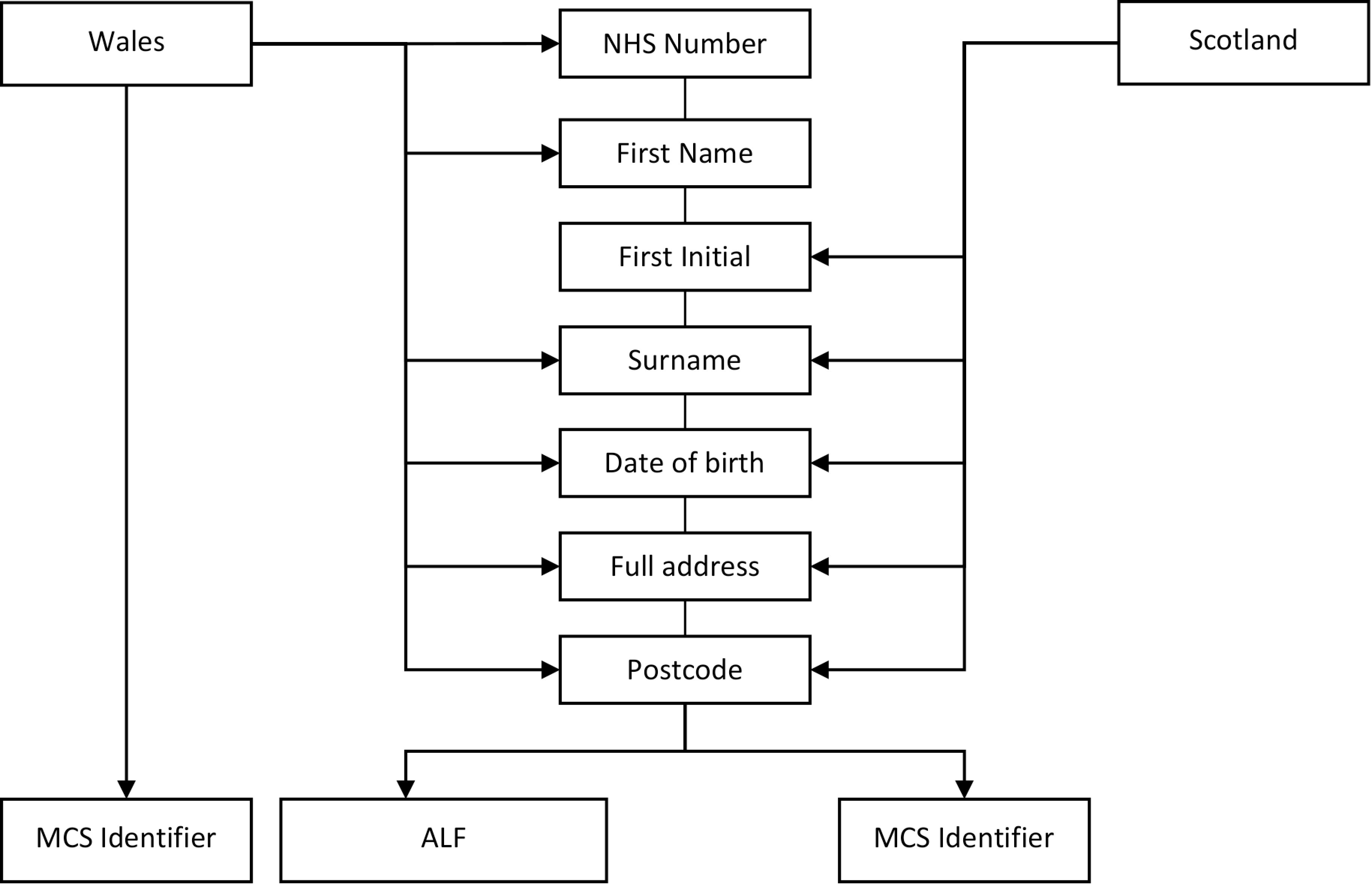
Observation: MCS Identifier is the anonymized field assigned by the Millennium Cohort Study team. ALF is the Welsh Anonymized Linkage Field assigned by the Welsh Trusted Third Party

Linkage for both sites is described in Figure 1 and is based on a combination of NHS identifier (NHS Number or CHI), all or part of the first name, surname, date of birth, address and postcode of residence.

Compared with the previous linkage to mother’s maternity hospital record and cohort member’s birth record [14], linkage for this project relied more on the cohort member’s details and less on the mother’s details.

#### Transfer to and linkage within SAIL

**Figure 2: Data governance and flow diagram.**
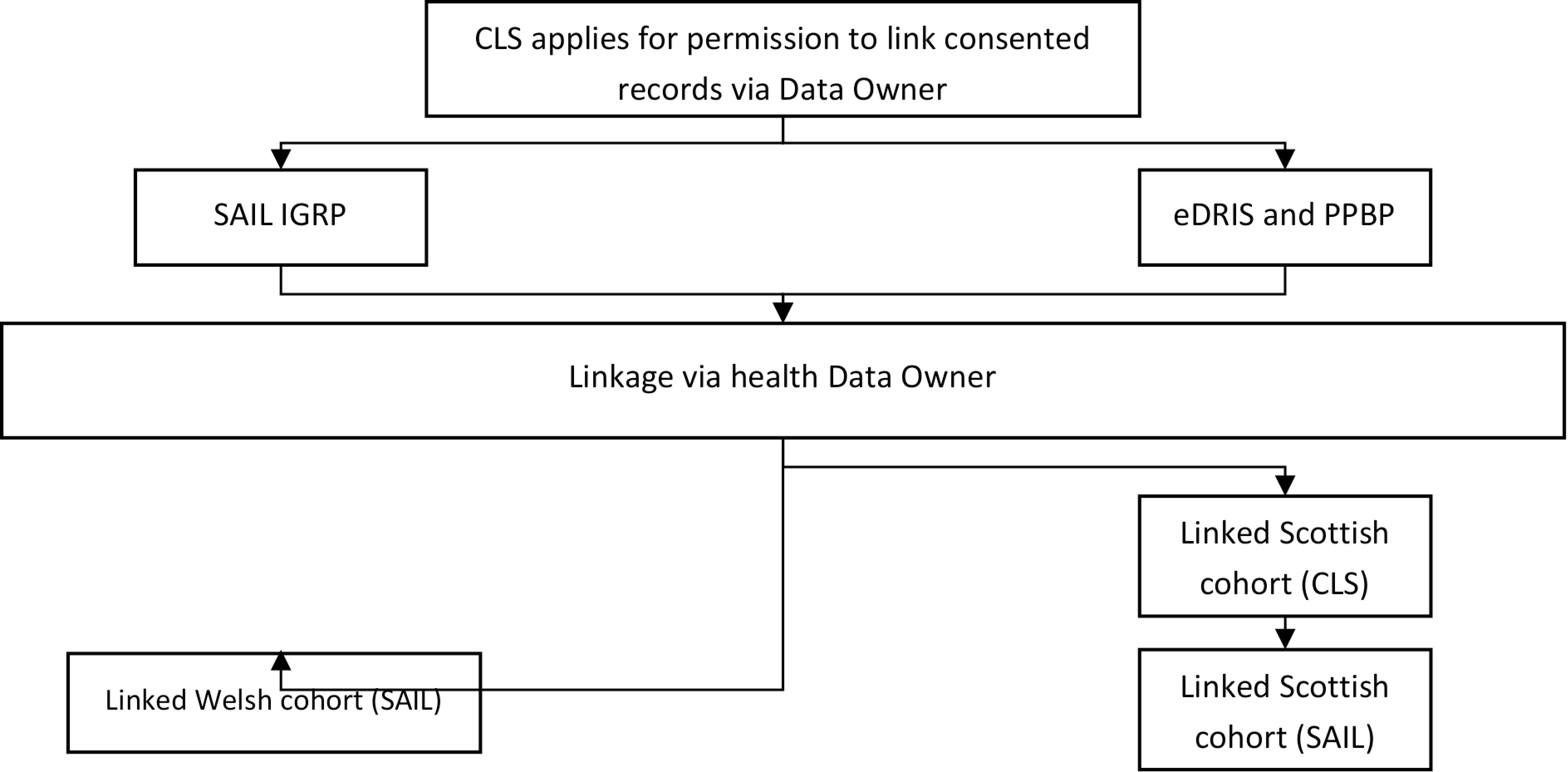
Observation: CLS = the Centre for Longitudinal Studies; SAIL IGRP = the ethics board for projects wishing to use the SAIL databank and Welsh linked data; eDRIS and PPBP = ethics boards for projects wishing to use Scottish routine health data

For linkage purposes, both TTPs assigned identifiers common to the MCS and routine health data sources. In Scotland, health data from cases matched on the CHI number were transferred directly to CLS with the CHI number replaced with the project-specific MCS identifier. These data were then securely transferred to the SAIL Databank and provisioned to the project. The data permissions and flow process is shown in Figure 2.

NWIS uses an encrypted Anonymous Linkage Field (ALF), based on the symmetrical block cipher Blowfish algorithm [20], for all linked datasets. Welsh health data are stored centrally under appropriate permissions and provisioned following approval by the local Information Governance Review Panel and CLS MCS application based on the ALF linkage already being completed, unless new data are needed for a project. A look-up table maps between MCS and health datasets using both ALF and MCS identifier.

### Study cohort representativeness

The UK MCS cohort includes Welsh and Scottish cohorts because it was not possible to exclude these from the full analyses, given the subset of data available for this project. The Welsh study sample included here comprises 13.3% of the UK MCS cohort, and the Scottish, 10.3%. The MCS cohort is itself not fully representative of the general UK population, being over-representative of children from lower socio-economic areas [3].

Because the sub-population sizes are so different, all figures relate to percentages, rather than household size, which is reported as the mode for each sub-population. Percentages were weighted using survey and non-response weights to account for the clustered sampling, attrition between contacts, and consent to data linkage [21].

Given the subpopulations are dependent, and the data are proportional in nature, we were not able to perform statistical tests to measure the degree of difference between these populations.

## Results

### Acquiring the datasets

Linkage applications were approved for Wales and Scotland, but were declined for England after 2 years, owing to concerns about the wording of the consent forms. Hence, the remainder of this report focuses on experience of linkage for Scotland and Wales only.

For Scotland, the original application was submitted in March 2015, approved in July that year, with data received by CLS in May 2016. The majority of the process involved linking the cohort to their health records. Once linked data had been released to CLS, a second application was required to share the data with the project team, which took one month.

For Wales, obtaining data took approximately 3 months, as the data were already linked and held within SAIL, and work could commence processing and analysing the datasets as soon as IG approval had been obtained in April 2015.

### Participants

**Figure 3: Flow chart showing inclusion criteria and numbers for the final linked population:**
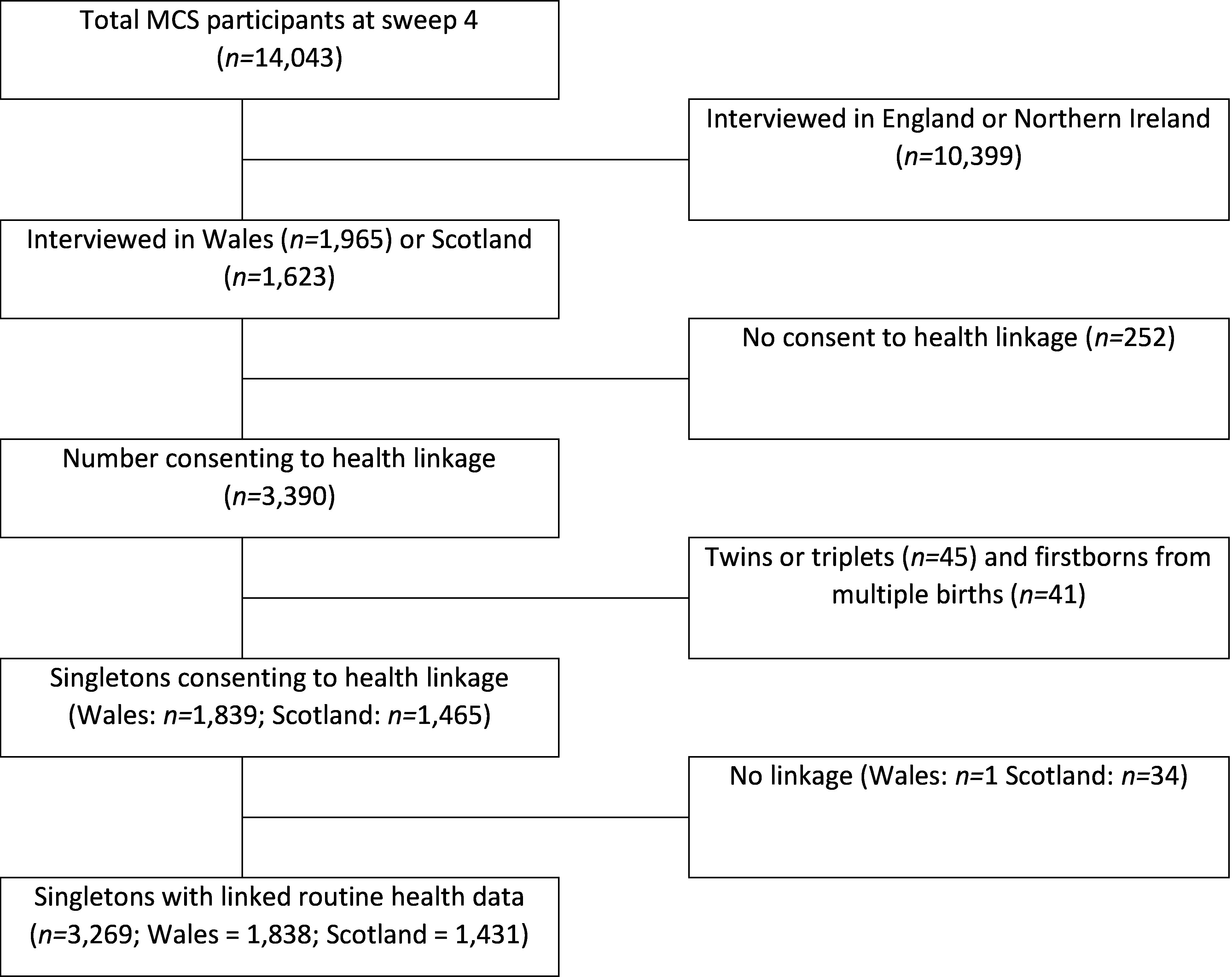
Observation: MCS = Millennium Cohort Study

Of the original 18,552 families interviewed at MCS1, 2,760 were interviewed in Wales, and 2,336 in Scotland. At MCS4, 1,965 families (with 1,951 singletons) were interviewed in Wales and 1,623 (1,598 singletons) in Scotland. After excluding those families who moved out of the UK, non-singleton children, those without consent to link health data, and the English and Northern Irish cohorts, the overall baseline study population was 1,838 Welsh children and 1,431 Scottish children (see Figure 3).

Linkage was successfully completed for almost all children for whom consent had been given. In Wales, 99.9% of children were matched to WDS and in Scotland 97.6% of those sent to ISD were matched to CHI.

Because linkage was conducted on population spines rather than for individual datasets, with unique identifiers then mapping across multiple data sources, separate specificity and sensitivity analyses were not required. We were not able to measure population-level coverage because, although we had access to data for all Welsh children, we only received Scottish data for the linked cohort.

Table 2 gives the number of children with at least one record in each dataset. For Wales, child health and GP datasets had the highest proportion of records, at 99.6% and 83.6% of the project baseline population, respectively. The Scottish child immunisations dataset contained 100% of the project population, but linkage was lower for the general Scottish child health dataset (91.3%). It appears that the Scottish immunisation dataset contains records on children who do not receive any immunisations, whereas the Scottish child health dataset only contains information on children who have interactions with the service. To avoid confusion with the Scottish child health dataset, Table 2 omits the separate Scottish immunisations dataset.

**Table 2: Family, maternal, and child characteristics of the sample (N=162,847) across child health groupings:** 

Dataset setting	Wales (N = 1838)	Scotland (N = 1431)

Child Health (excluding immunisations)	1831 (99.6)	1306 (91.3)
Emergency Department	1200 (65.3)	1033 (72.2)
Hospital inpatient	1334 (72.6)	833 (58.2)
Primary Care General Practice	1537 (83.6)	Not available

Inpatient records were present for 72.6% of the Welsh cohort, and 58.2% of the Scottish cohort. Sixty-five percent (65.3%) had attended a Welsh, and 72.2%, a Scottish, Emergency Department at some point between their 9th and 14th birthdays.

### Study cohort representativeness

Table 3 outlines general demographic details for the linked sub-populations compared with the full MCS cohort. Percentages for cohort member gender and main respondent at birth of cohort member are from MCS1 responses. Otherwise, the results refer to MCS4.

**Table 3: Demographics for the linked and total Millennium Cohort Study populations.** Observations: MCS = Millennium Cohort Study; NCCHD =National Community Child Health Dataset; GP = Welsh Longitudinal General Practice dataset; EDDS = Emergency Department Data Set; PEDW = Patient Episode Database for Wales; Child Health = Child Health Systems Programme; AE 2 = Accident and Emergency version 2 dataset; SMR01 = General Acute Inpatient and Day Case – Scottish Morbidity Record; CM = cohort member; SES = Socioeconomic status.

	All	Wales					Scotland			
		
	MCS	MCS	NCCHD	GP	EDDS	PEDW	MCS	Child Health	AE2	SMR01
**Number in MCS4**	13857	1838	1834	1537	1200	1334	1431	1306	1033	833

**Demographics and descriptives (% unless otherwise indicated)**										
**CM Gender (MCS1)**										
Male	50.7	53.0	52.9	53.5	56.8	56.3	50.2	50.4	52.3	53.3
Female	49.3	47.0	47.1	46.5	43.2	43.7	49.8	49.6	47.7	46.7
**Main respondent age at birth of CM (grouped, MCS1)**										
12-19	8.6	8.8	11.3	11.7	13.5	13.2	8.5	11.9	11.7	12.9
20-29	46.8	47.5	49.5	49.4	50.1	49.0	41.3	43.2	47.0	46.1
30-39	42.5	40.7	37.6	37.6	35.4	36.5	46.3	42.7	39.0	38.9
40+	2.2	2.9	1.5	1.3	1.1	1.3	3.9	2.1	2.3	2.1
**Mode of household size**	4	3	3	3	3	3	3	3	3	3
**Main respondent relationship to CM (MCS1)**										
Natural mother	99.7	99.8	99.7	99.7	99.6	99.7	100.0	99.7	100.0	99.5
**Main respondent relationship to CM (MCS4)**										
Natural mother	96.6	98.0	97.8	97.7	97.2	98.0	100.0	100.0	100.0	100.0
**CM ethnicity**										
White	83.7	97.2	97.2	97.4	97.0	97.2	97.6	97.3	97.3	97.6
Other	16.3	2.8	2.8	2.7	3.0	2.8	2.4	2.7	2.7	2.4
**Parents/Carers in the household**										
Both natural parents	72.1	66.3	66.4	65.9	63.3	65.2	70.3	69.6	69.2	67.7
Natural mother and step-parent	5.0	6.8	6.8	6.9	7.0	6.7	6.8	6.6	6.9	7.9
Natural mother only	20.2	23.5	23.5	23.6	25.5	24.9	21.1	22.0	22.2	23.2
Other	2.6	3.5	3.3	3.6	4.2	3.2	1.8	1.8	1.7	1.2
**SES**										
Management and Professional	28.8	23.6	23.6	22.8	22.3	21.8	23.4	24.1	22.5	19.0
Intermediate	17.4	11.8	11.9	12.1	12.7	11.3	14.8	14.4	15.4	15.7
Small employer and self-employed	7.1	5.0	5.0	5.2	5.0	5.5	4.7	5.0	3.8	4.9
lower supervisory and technical	4.4	3.1	2.9	2.9	2.8	3.5	3.1	2.6	3.2	3.3
Semi-routine and routine	33.8	18.8	18.8	18.4	19.6	18.5	19.2	18.9	19.5	19.9
Not applicable	8.5	37.7	37.8	38.6	37.5	39.4	38.8	35.1	35.5	37.3

Overall, the Welsh and Scottish MCS populations are not noticeably different from the UK MCS cohort, nor were the linked health data populations different from the national Welsh and Scottish consenting MCS populations. The Welsh and Scottish populations had fewer cohort members from non-White ethnic groups, and the main respondent was less likely to have semi-routine or routine job and more likely to be in a “Not applicable” employment category. For socio-economic status, “Not applicable” includes unemployed, full-time students, and unstated and unclassified employment categories.

The Welsh and Scottish cohort members were similar to the UK MCS cohort in terms of gender (53%-50.2% boys, respectively, compared with 50.7% boys in the UK MCS), and the linked routine data populations were not markedly different from the national base populations. However, there were more boys linked to the emergency department and inpatient datasets for both Wales and Scotland than in the base MCS populations (56.8% and 56.3% compared with 53% for Wales, and 52.3% and 53.3% versus 50.2% for Scotland).

Scottish cohort members are more likely to live with both natural parents (70.3%) than the Welsh cohort (66.3%), but less likely than the UK MCS cohort (72.1%). Welsh cohort members are more likely to live with their natural mother only, or to live in another family type. The mode household size was similar among all sub-populations.

For all sub-populations, the main respondent was overwhelmingly the natural mother at both MCS1 and MCS4 (MCS1: 99.7%-100%; MCS4: 96.6%-100%).

Welsh main respondents were more likely to give birth to the cohort member when aged less than 30 years (56.3%) than those in the Scottish (49.8%) or UK MCS (55.4%) cohorts. Scottish main respondents were more likely to be 30 years of age or older at birth of the cohort member (50.2%) when compared to Welsh (43.6%) or UK MCS (44.7%).

All of the linked populations showed higher levels of young mothers (aged 12-29) and lower levels of older mothers (aged 30+) than the national base populations.

## Discussion

### Key results

Linkage of demographic details on NHS health registers to MCS cohort participants by NHS Trusted Third Party organisations in Scotland and Wales was feasible and was completed for virtually all children. While the Welsh data were obtained quickly, owing to the availability of linked data in the SAIL databank, delays in acquisition of Scottish data, and non-acquisition of English data, is consistent with the experiences of others [7, 2]. That delays in acquiring data has been reported by multiple countries suggests that this is a broader research issue requiring further guidance.

Linkage matching of health datasets was higher than the 92% for both Wales and Scotland reported in the birth registration study [14]. Due to the nature of the data received, we are not able to test completeness of linkage for these specific datasets. We only received health data for the linked Scottish cohort, for example, and not for the full Scottish or Scottish child population. We are therefore unable to measure linkage rates for individual datasets. However, as linkage was conducted on population spines for both countries, with the same unique identifiers being used to record individuals in different data sources, and linkage was high for these population spines, we propose that linkage rates are likely to represent health service use rather than linkage success. Not every child will have had a hospital inpatient or emergency department episode and so will not appear in these datasets. Population-based health service usage rates are poorly reported in the literature, making this difficult to measure. However, the health services through the NHS are free to access, meaning that the population is unlikely to have financial restrictions on healthcare [22]. A report by the Nuffield Trust found that children living in more deprived areas of England are more likely to attend hospital emergency departments and to have more preventable emergency hospitalisations (51.5% of the population) than those from less deprived background (32.6%) [23]. These findings are lower than our linkage rates, but our inpatient subpopulations were not restricted to preventable emergency admissions.

As expected, virtually all children had records in the Welsh child health dataset, as this dataset contains information pertaining to the child’s birth, development and immunisations. The Scottish child immunisations dataset contains a record for each child, even those with no immunisation records, whereas the Welsh immunisation dataset only contains records where the child has been given at least one immunisation.

The lower number of retrieved records in the GP data is likely to reflect the percentage of GP practices contributing data to SAIL, and that not all healthy children will have attended a GP [24]. Most of the Welsh children had GP records (83.3%). This is higher than overall SAIL coverage of 78% and is likely due to partial coverage of records when patients move between SAIL and non-SAIL data-providing practices. Accurately describing GP coverage is a challenge as the system is dynamic, with practices being created, merged or closed. Hence, unless there is complete coverage of GP systems, the GP record will be partial for some of the cohort.

Demographically, our linked study population of consenting, linked singletons do not noticeably differ from the entire MCS population in many aspects, which suggests a relatively representative sample within the MCS cohort. This is particularly relevant for the disorder-specific aspects of our study (see, to date, [9][10]). The subpopulations for the different linked health datasets were also not markedly different from the base study population.

Our sample has a greater number of white respondents compared with the full MCS population, reflecting the sampling design and the fact that most people from black and minority ethnic groups reside in England, and has a higher proportion of people in uncategorised employment main respondents. Wales and Scotland have overall White British populations of 97.6% and 96% respectively, compared with 93.26% in England [25, 26].

Our sample also contains a higher proportion of children whose parents are not employed or who are in a “not stated” employment category. As the MCS cohort was deliberately chosen to over-represent more deprived areas, care must be taken when applying research findings from this linked cohort to the wider population, especially in relation to emergency admissions [23]. Our findings of higher rates of younger mothers than the base populations may also reflect higher rates of deprivation in Wales and Scotland, as deprivation has been found to be both a factor leading to, and an outcome of, young motherhood [27].

While there were no differences between the Welsh and Scottish base MCS cohort and the linked health dataset populations, there were noted differences in gender and maternal age, especially for inpatient and emergency department datasets. Several studies have found male children to be more likely to receive healthcare than female children, especially in inpatient and emergency departments [28-30]. This disparity has been speculated to be due to both cultural, physiological and behavioural differences in gender. Piccini et al reviewed several studies that found boys appeared to have preferential access to healthcare in non-Western cultures [29]. Both Piccini et al and Hon and Nelson [28] reported differences in rates of disease among boys and girls, although it is not always the case that boys have higher rates of disease. McQuinn and Campbell found gender-related emergency department attendance to be related to the child’s choice of sporting activity, with boys tending to play more contact sports than girls [30]. It is, therefore, not unsurprising that our emergency department and hospital inpatient subpopulations have higher rates of male children than female children. If anything, our findings of 53.3% and 56.3% boys for Scottish and Welsh inpatient datasets respectively are lower than Hon and Nelson’s average of 59% boys. These findings will be explored further in health-specific research within this project.

Data from routine sources for consenting cohort members in a longitudinal survey were retrieved for between 70% and 99% of participants, depending on the dataset. Given that the cohort comprised children under 14 years of age, it is perhaps not surprising that linkage to datasets relating to childbirth, pregnancy, early years, and immunisations had the highest yield of retrieved records.

Several studies have reported biases in relation to survey consents, with certain demographics more likely to give consent than others [6]. Consent to linkage for MCS participants was sufficiently high to not make this an issue, although, in performing our analyses, we have used the consent weights developed by Sera et al. [21]. However, as with other studies, ours was hampered by the complexities of consent as interpreted by different data owners.

Differences between the structure and content of routine datasets from different UK countries and how these were harmonised in order to create comparable explanatory and outcome variables for research will be covered in a separate paper.

Currently, linking routinely collected data to survey data requires informed consent from participants. It can be difficult to future-proof consent forms, despite best efforts using available governmental guidance [31]. Securing enduring consent against a changing information governance landscape is challenging, as the current standards at the moment when consent is obtained may not be acceptable at later stages. Despite the rise in research using large linked datasets over the last decade, uncertainty remains regarding how to ensure adequacy of consent to link to other data sources and whether this is consistently interpreted. This is particularly apparent in the disparities we experienced in approvals to link the health data between each UK country. While this confusion may be resolved when the MCS cohort is re-consented at the next sweep, the lack of consistency between data custodians can present a significant obstacle to successful completion of funded projects. It could be argued that ignoring participants’ expressed wishes to have their data linked for research would be detrimental to public perceptions of research [32]. More research is needed to determine public attitudes for consent to link routine data to survey responses, especially in the case of longitudinal child studies when children whose parents gave consent become able to consent themselves [33, 34].

This study builds on the previous MCS linkage work, both in validating linkage consistency, and, more importantly, supplementing the amount and type of linked data to this cohort. Linkage to longitudinal routine health records has the ability to provide a rich research resource for further studies. The resulting linked cohort has been used to better understand timeliness of childhood vaccination [9] and comparing GP-rated versus maternal-reported history of childhood wheezing [10].

### Limitations

The amount of data expected but not acquired from NHS Digital yielded less statistical power than one corresponding to a large study population. Thus, some results are only descriptive, although they are generalisable to Wales and Scottish populations and incorporate survey weights to account for attrition and sampling design. An attempt to access linked English hospital data will be made at a later stage once agreement has been reached between CLS and NHS Digital. CLS are currently obtaining consent for linkage of health records at the age 17 MCS sweep (MCS7). Re-consenting the MCS cohort will enable longer-term follow-up.

Lack of access to the non-consenting MCS population means that we are unable to look at differences between the different groups of linked-consenting, unlinked-consenting, and unlinked singleton births. However, this could be an area of further research. The MCS team have published a report on PEDW linkage using the full consenting dataset [35], but more research is needed to compare the different groups in order to better understand the linked data as a research resource.

Similarly, while our sample is largely representative of the wider MCS cohort, and of local ethnicities, it is not clear how this linked cohort differs from the wider Welsh and Scottish population. Unfortunately, it was not possible to compare linked and unlinked populations as part of this project, but we would recommend this as a useful area of future work.

### Lessons learned

Our study shows that linking national cohort responses to routine health data across multiple jurisdictions has the potential to create large and complex research datasets. However, based on our experiences, we would recommend that researchers wishing to create new datasets from previously unlinked data sources obtain approval in principle from the data owners prior to starting the project. While we would advocate for the reuse of previously-linked data sources for future research (pending approval by the data owners and ensuring consent is respected), obtaining approval to link new data sources can be time-consuming beyond the project timeframe. Some data sources, such as NHS Digital in England, publish minutes from their ethical approval panels. We would advise researchers, in the early stages of project development, to familiarise themselves with the types of projects that have been approved by their chosen data sources. This may identify potential delays if the proposed data use has not previously been approved. It would also be advisable to have representation from data owners on the project Steering Committee.

Harmonising the health data from different countries is sufficiently detailed to be tackled in a separate article. We would, however, recommend that researchers using data sources from similar settings over multiple countries familiarise themselves with the metadata for each, and include harmonisation as a pre-analytical process in their work plan.

## Conclusions

Our project has found that linking cohorts to routine health data is challenging but worthwhile, as the linkage rates are high enough to potentially provide valuable additional research information. The linkage produced for the project have already been used to measure childhood immunisations and respiratory conditions, with reports from research into injuries and physical activity in progress. The linkage enables both additional information and the opportunity to validate the different data sources against each other, thus providing both enriched data and methodological rigour.

However, there are issues around inconsistent handling of consent between data providers, and in the length of time taken to acquire the data. Until these issues are addressed, researchers should consider these potential delays when planning their projects, and data custodians could look to proactively acquiring datasets for research use.

Linking survey and routine data is a useful research tool. As with issues around consent, greater consistency between distinct but related data owners both regarding access to, and use of, the data is needed by the research community and wider public in order to make full use of the potential linked cohort and routine data can offer to researchers.

## Other information
